# On the origin of the nucleus: a hypothesis

**DOI:** 10.1128/mmbr.00186-21

**Published:** 2023-11-29

**Authors:** Buzz Baum, Anja Spang

**Affiliations:** 1 MRC Laboratory of Molecular Biology, Cambridge Biomedical Campus, Francis Crick Avenue, Cambridge CB2 0QH, UK; 2 Department of Marine Microbiology and Biogeochemistry, NIOZ, Royal Netherlands Institute for Sea Research, 1790 AB, Den Burg, Texel, The Netherlands; 3 Utrecht University, 3508 TC, Utrecht, The Netherlands

## Abstract

In this hypothesis article, we explore the origin of the eukaryotic nucleus. In doing so, we first look afresh at the nature of this defining feature of the eukaryotic cell and its core functions - emphasizing the utility of seeing the eukaryotic nucleoplasm and cytoplasm as distinct regions of a common compartment. We then discuss recent progress in understanding the evolution of the eukaryotic cell from archaeal and bacterial ancestors, focusing on phylogenetic and experimental data which have revealed that many eukaryotic machines with nuclear activities have archaeal counterparts. In addition, we review the literature describing the cell biology of representatives of the TACK and Asgard archaea - the closest known living archaeal relatives of eukaryotes. Finally, bringing these strands together, we propose a model for the archaeal origin of the nucleus that explains much of the current data, including predictions that can be used to put it to the test.

## 1 Introduction

**Figure 1:**
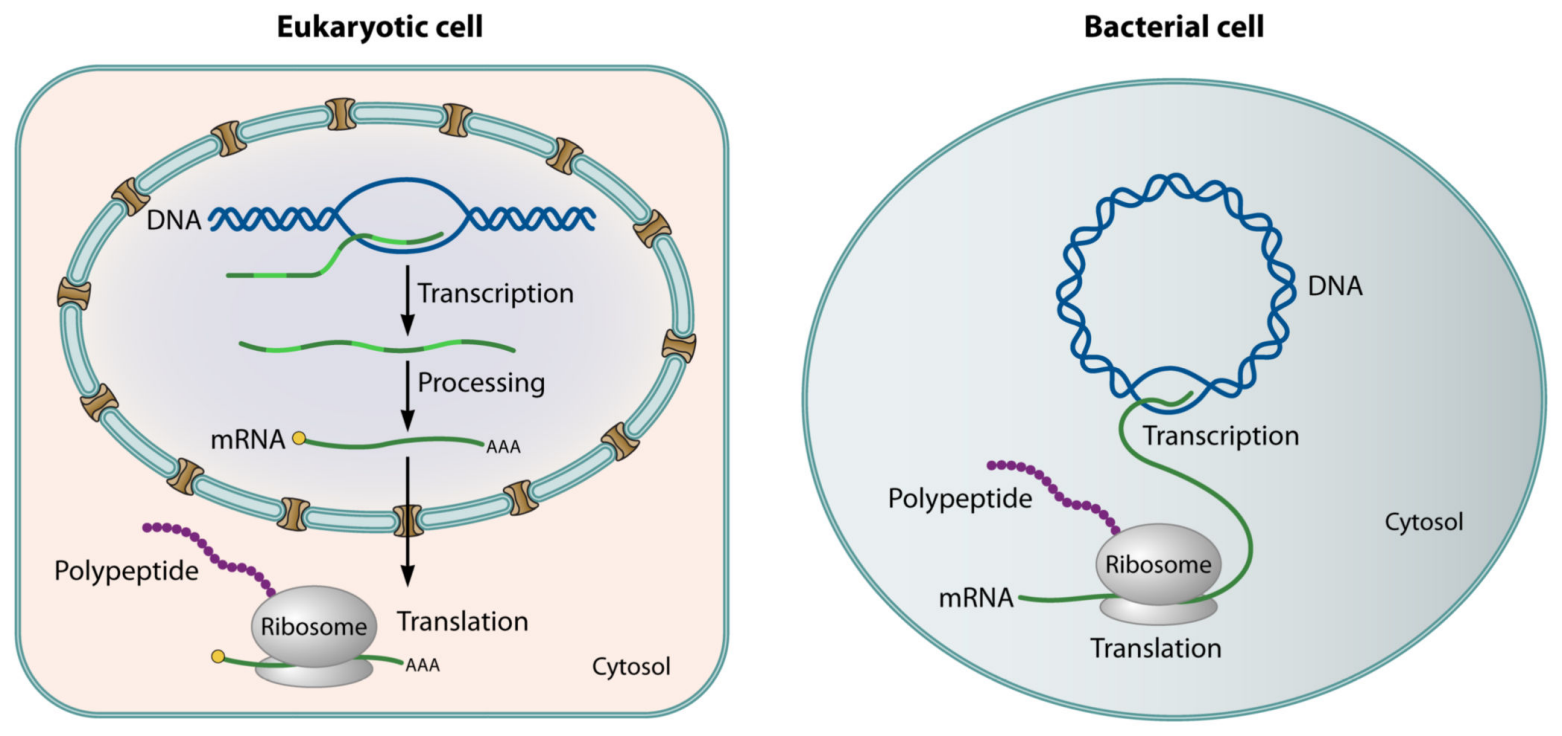
These diagrams compare the spatial organization of gene expression in a schematic eukaryotic and bacterial cell. The images emphasize that, while transcription and translation are separate in eukaryotic cells, they are coupled in many bacteria. A key question posed by this article is whether or not transcription and translation are strongly coupled or partially uncoupled to enable the local translation of some transcripts in close archaeal relatives of eukaryotes.

**Figure 2:**
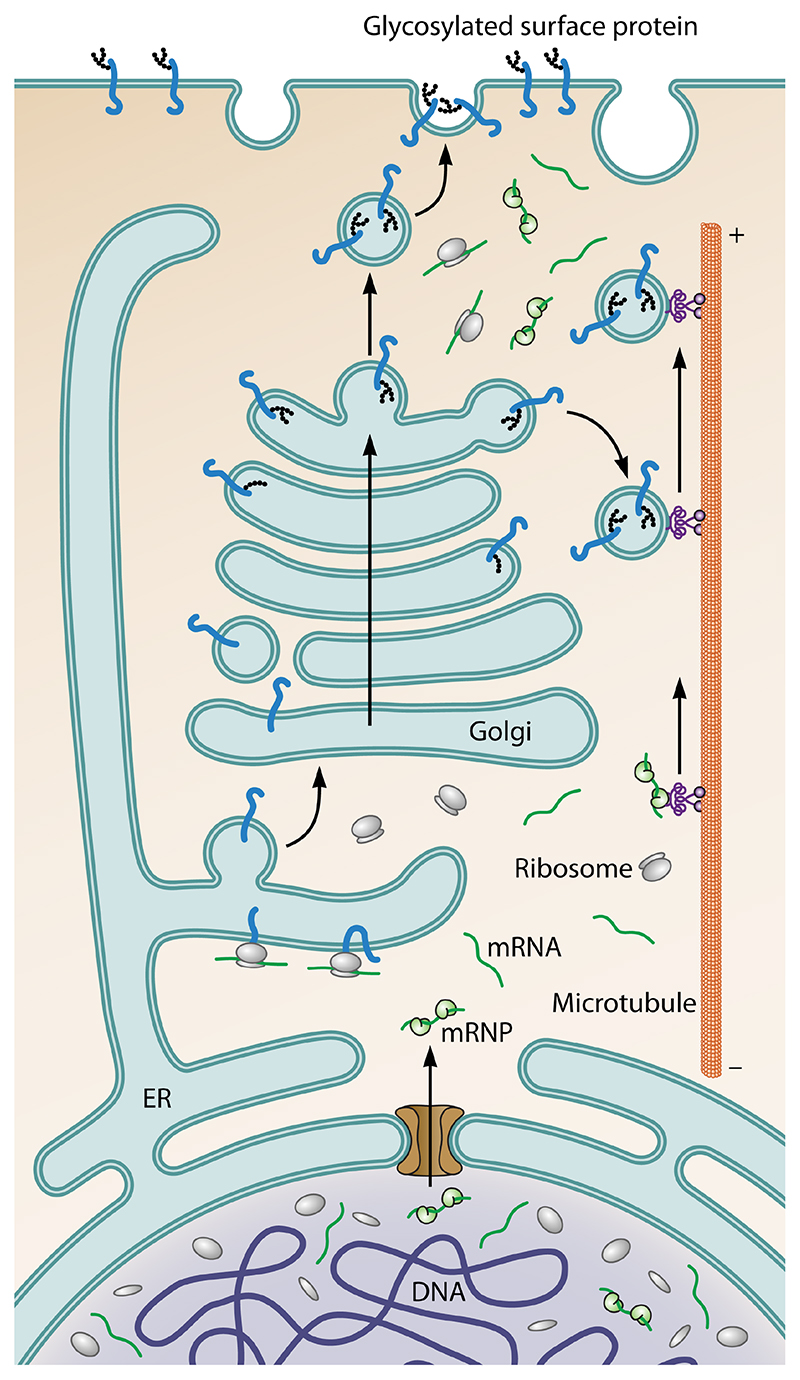
Diagram shows the path of genetically encoded information in eukaryotic cells as it moves out of the nucleus (bottom) towards the cell periphery (top). DNA is transcribed in the nucleus. The RNAs generated are then processed and exported through nuclear pores into the cytoplasm. While many messenger RNAs (mRNAs) are rapidly translated upon entering the cytoplasm, others remain inaccessible to ribosomes as the result of RNA-binding proteins. Some of these are trafficked along microtubules in an inactive state, enabling local protein synthesis in response to signals, e.g. at the tips of axons. In parallel, proteins carrying signal peptides which are translated by ribosomes at the surface of the rough endoplasmic reticulum (ER), move through the ER and Golgi, where they are modified by glycosylation, packaged into vesicles, and trafficked out to the cell periphery along microtubules.

The nucleus is the defining feature of eukaryotic cells. All eukaryotes have one and its presence marks them out as different from the vast array of bacteria and archaea (Figure 1). While the average bacterial or archaeal cell is structurally less complex than eukaryotic cells (with notable exceptions [1–4]), all eukaryotes possess an elaborate maze of internal membranes that extend outwards from the centrally positioned nucleus. Sitting at the heart of the cell, the nuclear compartment acts as a safe haven for the genome. Beginning with transcription, splicing, and mRNA export, the directed flow of this genetically encoded information propagates outwards from the nucleus to the cell periphery (Figure 1 and 2). While all eukaryotes share this organization, it is not clear how it arose during evolution. In an attempt to answer this question, we bring together knowledge about nuclear structure and function, phylogenetic data, and recent cell biological studies in archaea. As we will see, the synthesis of this information is consistent with an archaeal origin for many nuclear activities, and leads us to propose a possible path for the gradual emergence of a separate nucleoplasm and cytoplasm early on during eukaryogenesis.

It should be noted that, while this article builds on ideas put forward in the inside-out model of eukaryogenesis [5], the model we propose here is distinct and more delimited in scope than the original model in that it does not attempt to explain the evolution of the eukaryotic cell as a whole. As a result, it is possible for elements of this model of nuclear evolution to be wrong without this invalidating the inside-out model. Conversely, certain elements of the inside-out model of eukaryogenesis could be erroneous without this having an impact on the validity of the model presented here on the evolution of the nucleus. Because this article focuses on the nucleus, it doesn’t include a comprehensive overview of the alternative models put forward to explain eukaryogenesis. While these are covered in brief (see Box 1), we would point readers interested in a detailed discussion of the topic to some excellent recent reviews on the subject [6–9].

## 2 Nuclear Architecture

To begin, we discuss the nature of the eukaryotic nucleus, distinct nuclear sub-compartments, the nuclear envelope, and the pores that connect the nucleoplasm and cytoplasm.

### 2.1 The nuclear-cytoplasm divide

**Figure 3:**
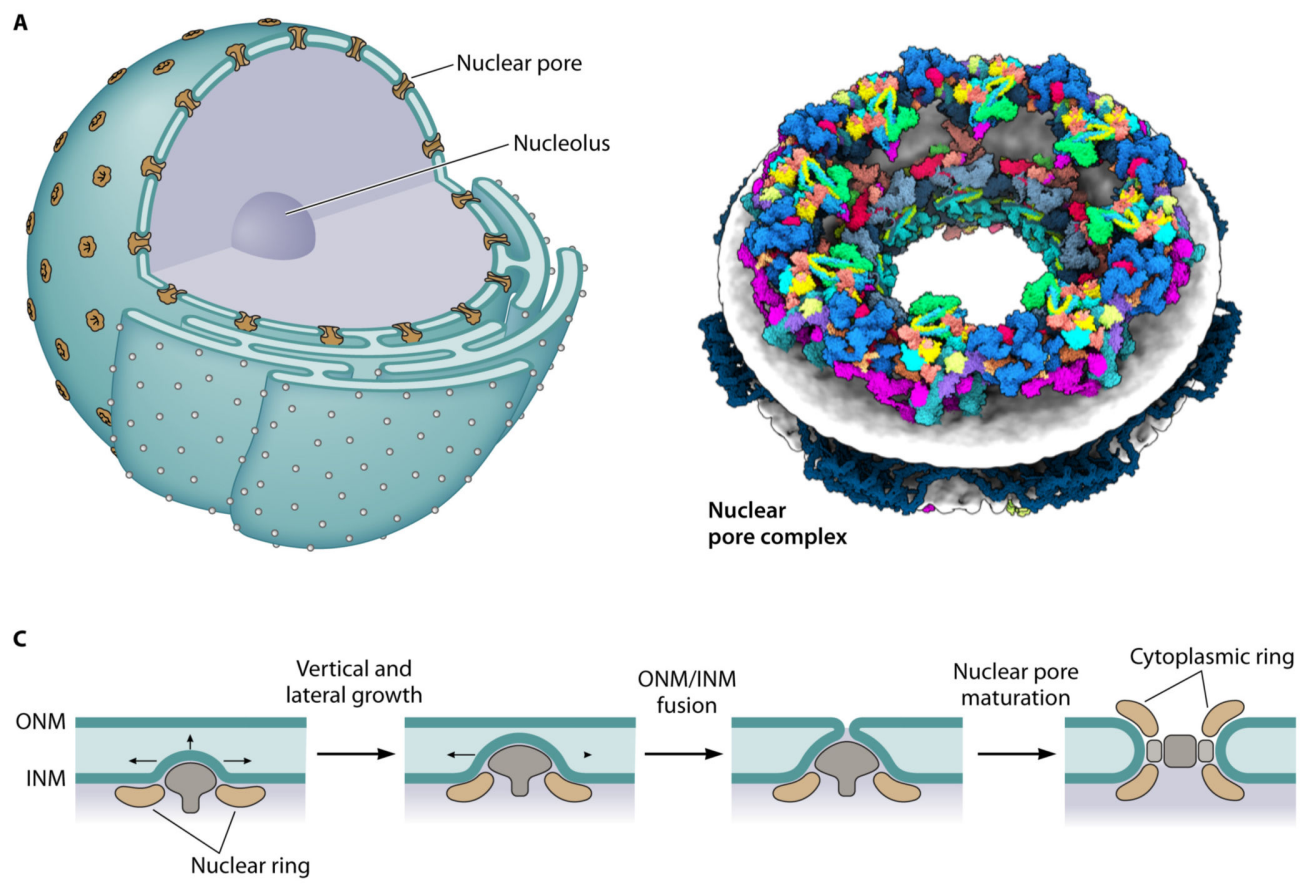
A. Diagram shows structure of the eukaryotic nucleus, and the endoplasmic reticulum (ER) to which it is connected. The nucleus is studded with nuclear pore complexes (NPCs). These NPCs sit at sites of high membrane curvature where the inner and outer nuclear membranes meet, and function as gated channels through which material can move between the nucleoplasm and cytoplasm. B. Image shows a single 8-fold symmetric nuclear pore complex inserted into the membrane viewed from one side (with kind permission of Agnieszka Obarska and Martin Beck). C. New nuclear pores are inserted into the nuclear envelope via two processes: i) insertion into gaps in the nuclear envelope as it reforms at mitotic exit and (ii) via interphase insertion. The diagram (adapted from Otsuke and Ellenberg, 2016, and based on electron microscopy data) shows a proposed path for interphase nuclear pore insertion from the inside out.

Because the nucleus appears to be enveloped by two lipid bilayers when imaged in cross-section using electron microscopy, it is commonly said to possess a bounding “double membrane”. This view is inaccurate though, because the inner and outer nuclear membranes are physically continuous (Figure 2 and 3A). The two bilayers are in fact connected to one another via numerous highly curved membrane connections or pores (Figure 3A and 3B). As a result, lipids are rapidly exchanged between the inner nuclear envelope and the endoplasmic reticulum (ER), without the need for protein channels like those required to transport lipids between the ER and the plasma membrane and mitochondria [10, 11]. Furthermore, the lumen of the ER and the space that lies between the inner and outer nuclear envelope form a single continuous fluid filled network that defines the nuclear boundary and extends throughout the eukaryotic cell (Figure 2 and 3A). In addition, because the nuclear envelope contains numerous pores, the nucleoplasm and cytoplasm should not be viewed as distinct compartments separated by a double membrane, but rather as sub-domains or discrete phases of a single compartment between which small molecules freely diffuse. In most eukaryotic cells, this mixing of the nucleoplasm and cytoplasm is accentuated at every mitosis following the loss of nuclear pores [11]. The continual exchange of nuclear material with the cytoplasm marks the nucleus out as very different from the other membrane-bound compartments within the eukaryotic cell. The contrast is clear when we consider non-nuclear organelles, such as endosomes, lysosomes, mitochondria and peroxisomes, which tend to have bounding membranes that function as physical barriers across which hydrophilic molecules cannot move unaided. As a result, each of these organelles is able to establish a unique internal milieu that is suited to its function and profoundly different from the surrounding cytoplasm. In the case of the lumen of the lysosome, for example, this includes a low internal pH (generated by active transport via pumps like the AV-type ATP synthase [12]), a high calcium concentration, an oxidizing rather than reducing environment, and limited pools of nucleotides [13]. Because of this, physical insults that perforate the bounding membrane of any one of these organelles can have catastrophic consequences for cell function, and often serve as triggers warning cells of pathogen invasion.

### 2.2 Sub-regionalization of the nucleus

At the same time, the nucleus is not structurally or biochemically uniform [16]. Like the cytoplasm, the nucleoplasm possesses functionally distinct domains [17]. It contains a nucleolus (Figure 3A), one of the first organelles to be described by cell biologists, where rRNAs are processed and assembled into pre-ribosomes [18]. Nuclei also contain near spherical Cajal bodies, sites of RNP processing and maturation, where telomerase and spliceosomal machinery are concentrated [16]. DNA replication [19] and repair [20] activities tend to be clustered in discrete foci in the nucleus. Transcribed and silenced chromatin have distinct nuclear locations [21]. Individual chromosomes have been reported to occupy their own territories [22]. And when perturbed, nuclei accumulate local stress granules [23] and, when infected, viral assembly factories [24]. Because these nuclear structures are not bound by membranes, they are likely established and maintained by weak multivalent interactions between proteins and/or nucleic acids. In some cases, these structures resemble vinegar droplets in oil, distinct liquid phases that some authors have termed “biomolecular condensates” [25]. Thus, even though nuclei are kept largely free of internal membranes during both interphase and mitosis [26], the nucleus is home to an ensemble of local sub-structures where different cellular components can be concentrated as and when required [25].

### 2.3 NPCs: gate-keepers controlling traffic between the nucleoplasm and cytoplasm

The exchange of material between the nucleus and cytoplasm is regulated by huge rivet-like nuclear pore complexes (NPCs) [27] (Figure 3B). These NPCs physically support the highly curved membrane at the sites at which inner and outer nuclear membranes meet, and are amongst the largest and most complex molecular assemblies in all of biology. NPCs are constructed from a set of ^~^500 proteins in yeast and ^~^1000 in humans, with a total mass of 50 or 120 megadaltons, respectively. The pore’s central scaffold is composed of two 8-fold symmetric rings stacked on top of one another (one facing the cytoplasm, the other the nucleoplasm). These symmetric rings act as a physical support for more peripheral proteins that sit at the cytoplasmic and/or nuclear face of the pore [28]. NPCs are anchored within the curved portion of the membrane by a small number of proteins that are physically embedded in the membrane. The NPC scaffold surrounds a central aqueous hole that has an internal diameter of ^~^50nm [29], which is enormous by cell standards. Thus, while chromatin, small vesicles, and most viral capsids cannot move between the nucleoplasm and cytoplasm, NPCs are large enough to allow the passage of some of the largest multicomponent molecular complexes present in cells, such as 25nm diameter pre-ribosomes. The transit of large proteins and protein complexes through the NPC is regulated by a central plug of disordered peptides rich in phenylalanine/glycine (FG) repeats [27]. By engaging in multiple low affinity interactions, these FG repeats generate a dynamic mesh that acts as a selectivity filter to limit the passage of macromolecules in a manner that depends on their size [30]. As a result, while small water-soluble molecules diffuse freely through the NPC between nuclear and cytoplasmic compartments, molecules or complexes greater than ^~^30 kDa in size, such as large proteins, RNPs, pre-ribosomes and mature tRNAs, only cross the nuclear/cytoplasm compartment boundary if recognized by importins or exportins. These carrier proteins alter the biophysical properties of the FG repeats that form the sieve at the center of NPCs to facilitate the passage of cargo to which they are bound [31]. Flux across the nuclear envelope has been estimated to be *~* 1 million macromolecules/second [31].

The association of cargo proteins with importins and exportins depends on the presence of specific Nuclear Localization Sequences (NLS) [32] and Nuclear Export Sequences (NES), respectively, and is regulated by the nucleotide status of an associated small GTPase called Ran (reviewed in [33]). Ran is loaded with GTP in the nucleus (by a chromatin-bound guanine nucleotide exchange factor or GEF), but switches to its GDP form in the cytoplasm as a result of the local stimulation of Ran’s GTPase activity by cytoplasmic GTPase activating proteins (GAPs). The physical separation of GEFs and GAPs sets up a gradient in Ran-GTP/Ran-GDP concentration across the nuclear/cytoplasmic compartment boundary that cells use to power the net, directed movement of large complexes either into or out of the nucleus, depending on whether the cargo is bound by importins (released by Ran-GTP) or exportins (released by Ran-GDP). It is important to note, however, that this Ran shuttle doesn’t require a membrane. All that is needed for Ran to polarize cellular space is the physical separation of GEFs and GAPs. In fact, Ran is used in exactly this way to polarise a single aqueous environment [34] following the loss of the nuclear/cytoplasmic compartment barrier in meiotic oocytes [35], as well as during mitosis in fertilized eggs and somatic cells [33, 36]. In these instances, the Ran-GTP/GDP gradient is used as a measure of distance [37, 38] to induce the nucleation of microtubules [39–41] and the remodeling of the cell cortex in the vicinity of RanGEF-bound chromatin [42–44]. More controversially, Ran, importin and a small number of nuclear pore proteins have also been implicated in the regulation of traffic across the diffusion barrier at the base of cilia [45], enabling, amongst other things, the local translation of cilia-targeted mRNAs. This suggests the possibility that some of the machinery used to govern the passage of molecules across the nuclear pore may have been re-deployed to regulate traffic into and out of cilia [5, 46].

Ran is not the only system controlling traffic across the nuclear pore. The Ntf2 system [31, 47] operates in parallel to Ran in many eukaryotes to support the unidirectional movement of mRNAs and pre-ribosomes from the nucleoplasm to the cytoplasm [48], which is one of the most important activities of the NPC [31, 47]. In this process, Ntf2 homologues help mRNAs that are part of large protein-RNA complexes, called mRNPs (Ribonucleoprotein particles containing mRNA), to cross the disordered mesh of FG repeats [49]. Ntf2 proteins are removed from these mRNPs by a family of ATP-dependent DEAD-box helicases that sit at the cytosolic face of the nucleus [50]. This renders the export process irreversible. A similar logic applies to mRNAs packaged as part of inactive mRNPs that are trafficked to peripheral regions of the eukaryotic cell, like the tips of neuronal processes and cilia. Again, this doesn’t require a compartment boundary. In these cases, mRNPs are silent when trafficked, but are disassembled when required by DEAD-box helicases at the cell periphery, enabling ribosome binding and protein synthesis at the appropriate time and place [51] (Figures 1 and 3).

### 2.4 NPC assembly and disassembly

Although NPCs are some of the most stable structures in the cell [52], they are removed from the nuclear envelope at every division. The extent, timing and location of mitotic nuclear pore disassembly varies widely across eukaryotes depending on the extent to which nuclear division is accompanied by a loss of the nuclear/cytoplasmic compartment boundary [11]). The loss of NPCs opens up transient holes in the mitotic nuclear envelope. In an “open” mitosis, say in a human cell, the entire set of NPCs are disassembled, leading to extensive mixing of the mitotic nucleoplasm and cytoplasm. By contrast, in a dividing fission yeast nucleus [53], a classical example of “closed” mitosis in which the nucleo-cytoplasmic diffusion barrier is retained during the process of nuclear division, NPCs are only disassembled at the center of the late anaphase nuclear bridge. This local loss of NPCs facilitates remodeling of the nuclear envelope and ER at the center of the bridge, allowing nuclear division to go to completion, yielding two daughter nuclei. Whatever the mode of division, to counter the loss of NPCs induced by rounds of mitosis and their dilution during interphase nuclear envelope growth, new NPCs must be continually inserted into the eukaryotic nuclear envelope (Figure 3C). NPC insertion occurs via two distinct pathways [54, 55]. First, nucleoporin sub-complexes are recruited into gaps left in the bounding nuclear envelope when the nuclear-cytoplasmic compartment boundary is re-established at mitotic exit [54]. During this process, ESCRT-III (Endosomal sorting complexes required for transport-III) proteins repair errors and seal holes that remain [56]. Second, during interphase, pores are inserted into growing nuclei in discrete steps from the inside-out [5], via a process that has only recently been studied in any detail [54, 55] (Figure 3C). This insertion begins with the out-folding of the inner nuclear envelope. Nascent nuclear pore components then accumulate at the neck of small membrane nuclear blebs, stabilizing their structure. The fusion of the bulging inner membrane with the overlying outer nuclear envelope then completes the process, generating a membrane-lined NPC that connects the nucleoplasm and cytoplasm. Finally, in some cells, like oocytes, which undergo extensive periods of growth, nuclear growth is fueled by the insertion of cytoplasmic membrane sheets termed annulate lamellae that contain pre-assembled pores [57]. In many eukaryotes, AAA-ATPase Torsin helps to power the membrane remodeling that accompanies NPC insertion [58]. While capturing snapshots of putative intermediates by electron microscopy has proven hard in typical eukaryotic cells imaged under physiological conditions, similar structures have been observed by electron microscopy under perturbed conditions across a wide range of eukaryotes [59, 60], implying that this process of pore insertion may be a generic and ancient one, as suggested by the inside-out model of eukaryogenesis [5].

## 3 What is the nucleus for?

Having reviewed nuclear architecture and the machinery used to control the exchange of material between the nucleus and the cytoplasm, we now discuss what might be considered the main functions of the nucleus. We can point to six, more or less distinct roles of the nucleus.

First, the eukaryotic nucleus provides a well-defined space in which to house and organize the genome. In eukaryotes, whose genomes (from 10Mb to >100,000Mb) tend to be much larger than those found in prokaryotes (0.5->10Mb), this requires extensive genome compaction, which is made possible by a large ensemble of abundant DNA-binding proteins.

Second, the eukaryotic nucleus protects the DNA from physical insults. In mechanically soft cells, like animal cells and *Dictyostelium*, the nuclear envelope is supported by an underlying lamina, analogous to the skull under our scalps, which prevents external physical forces from disrupting the information processing activities going on within the nucleus. Under moderate pressure, mechanical signaling in the nuclear envelope triggers an increased cortical contractility, which likely insulates the cell from external forces [61, 62]. If the mechanical resistance of the lamina has been overcome, the resulting rupture of the nuclear/cytoplasmic barrier allows antiviral defense systems concentrated in the cytoplasm, such as TREX-1, to leak into the nucleus, inducing DNA damage [63].

Third, the nucleus likely provides a chemically distinct and more reducing environment in which to house the genome. This is achieved, in part, through the exclusion of metabolically active organelles like mitochondria. This has the effect of limiting the exposure of DNA to genotoxic free radicals generated by respiration, perhaps helping to keep mutation rates within manageable levels.

Fourth, the eukaryotic nuclear envelope provides a physical boundary that separates transcription and translation. This distinguishes the core steps in eukaryotic gene expression from those described for most bacteria and some archaea [66, 67] - where the translation of genes frequently starts before transcription has finished (see [68] for an exception) (Figure 1). In eukaryotes, the physical uncoupling of these processes facilitates the processing of newly transcribed RNAs through the addition of a 5’ cap, a polyA tail, and intron removal [69], processes that take time. Quality control machinery ensures that mRNAs are only exported to the cytoplasm once processing is complete [48]. The spatial separation of transcription and translation also relies on the confinement of active ribosomes to the cytoplasm, through a carefully choreographed process of rRNA processing and ribosome assembly. This begins in the nucleolus where rRNAs are transcribed, processed and modified [70]. Note that rRNAs and tRNAs are processed via the removal of insertions and though modifications in bacteria and archaea as well as in eukaryotes [71]. Pre-ribosomes assembled within the eukaryotic nucleus are kept in an inactive state by proteins such as eIF6 [72]. Inactive pre-ribosomes are then exported from the nucleus through NPCs, so that the final steps of ribosomal assembly and activation can be completed in the cytoplasm, where mature ribosomal subunits encounter fully processed mRNAs, their substrates. In combination, these two activities, intranuclear mRNA processing and extranuclear ribosome assembly function to spatially and temporally uncouple transcription from translation in all extant eukaryotic cells. This has been suggested to augment the ability of eukaryotes to regulate gene expression, for example through alternative splicing [73].

Fifth, the nucleus functions as a coherent aqueous membrane-free space in which genomes composed of multiple chromosomes can be aligned and segregated. Since the forced association of mitotic chromatin with membranes has been shown to interfere with chromosome segregation [26], the transient loss of chromatin-nuclear envelope binding may be a functionally important part of the mitotic programme.

Finally, the partial separation of the nucleus and cytoplasm likely acts as a barrier to the replication of viruses entering the cell from the environment by enabling DNA nucleases to police the cytoplasm [74] and by sequestering away host machinery capable of replicating genomic DNA within the nucleus.

## 4 The origins of the eukaryotic endomembrane system

Having discussed the structure and function of the nucleus, in the following section we discuss the challenges of determining when the nucleus first emerged during evolution. In doing so, we look at recent phylogenomic data which suggest that eukaryotic cells likely arose as the result of a merger between an alphaproteobacterial cell and an asgardarchaeotal cell. We then briefly discuss the different types of models that have been proposed to explain the available data.

### 4.1 The absence of evidence

**Figure 4:**
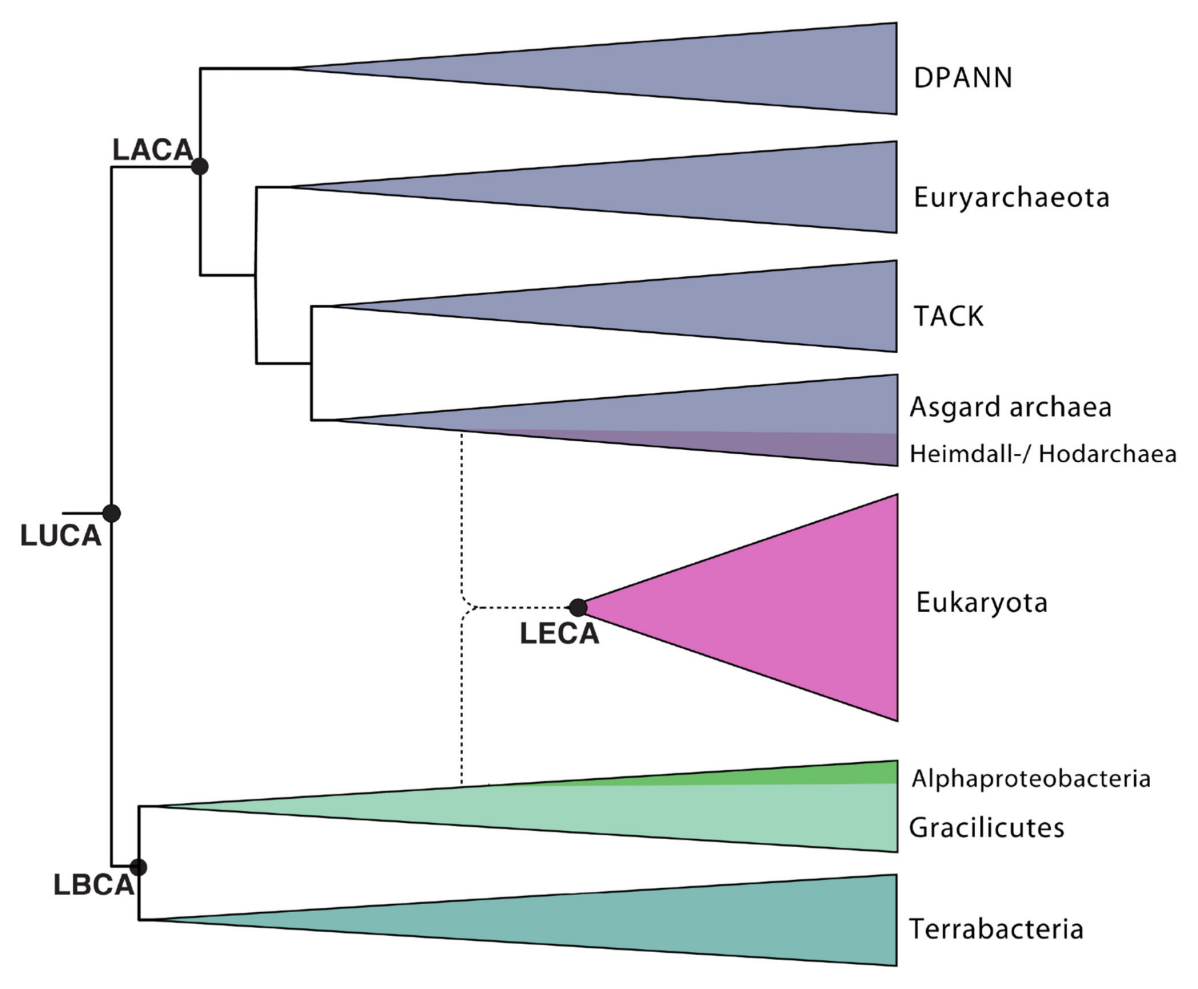
Schematic view of the tree of life with Bacteria in green, Archaea in purple, and Eukaryotes in pink. Labels on the tree mark the Last Universal Common Ancestor (LUCA), the Last Archaeal Common Ancestor (LACA), the Last Bacterial Common Ancestor (LBCA) and the Last Eukaryotic Common Ancestor (LECA). As depicted in the diagram, LECA is hypothesized to be derived from the merging of at least two partners: an alphaproteobacterial symbiont and an asgardarchaeotal host.

**Figure 5:**
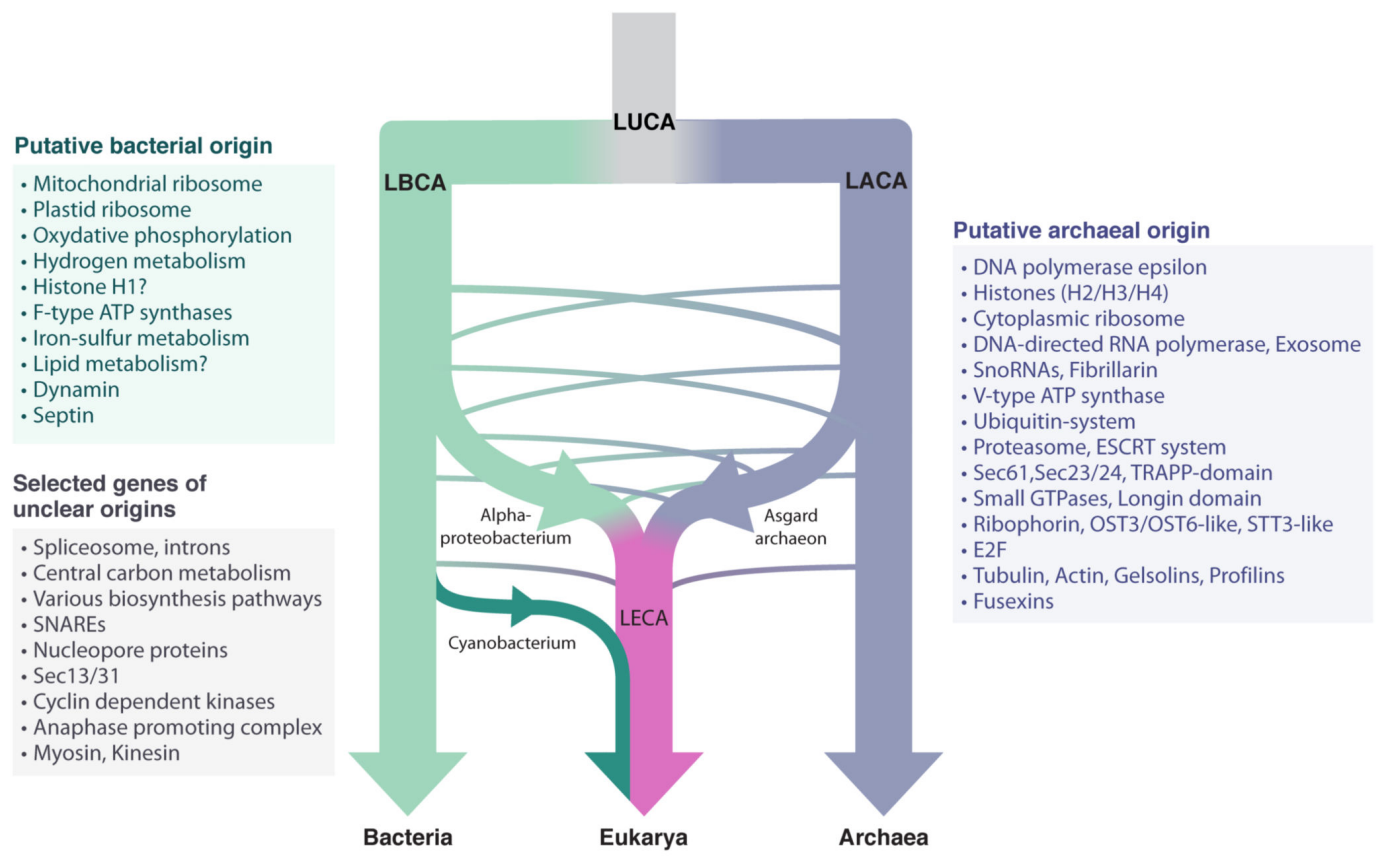
Schematic tree of life with Bacteria in green, Archaea in purple, and Eukaryota in pink - emerging from the merger of an alphaproteobacterial symbiont with an asgardarchaeotal host. Horizontal gene transfer, which can complicate the phylogenetic analysis, is indicated by interconnecting lines. The light-shaded boxes indicate enzymes of likely archaeal (purple), bacterial (green) or unknown (grey) origin. Question marks indicate putative origins that are less clear.

While the nucleus plays an indispensable role in the life of all eukaryotic cells, its origin and the timing of its emergence relative to other eukaryotic traits remain unclear. The challenge of establishing a precise order of events that led to the formation of a eukaryotic cell with a nucleus and a complex endomembrane system in part reflects the lack of “simple eukaryotes”, i.e. direct descendants of intermediates along the path towards the formation of today’s complex eukaryotes that have remained relatively unchanged over long periods of evolutionary time. Diplomonads like *Giardia* were once thought to be an early diverging “primitive” eukaryotes of this type, because *Giardia* cells lack canonical mitochondria and appear to have a simplified endomembrane system with a single internal membrane (including a continuous inner and outer nuclear envelope, ER, Golgi and endocytic/lysosomal compartment) [75]. More recently, however, *Giardia* has been shown to possess mitochondrial-like organelles, referred to as mitosomes [76, 77]. Thus, if aspects of *Giardia* cell biology can be justifiably described as simple, this simplicity is likely a consequence of evolutionary streamlining - as has been shown to be the case for other microbial eukaryotes, including in the oxymonad *Monocercomonoides sp*., which has lost mitochondria altogether [78]. Thus, as far as we know, all extant eukaryotic lineages emerged from a complex Last Eukaryotic Common Ancestor (LECA) [79, 80] that had all the hallmarks of a modern eukaryote, via an explosive evolutionary radiation sometime between about 1 and 2.3 billion years ago [81–83] (Figures 4 and 5). The absence of architecturally simple eukaryotes situated as sister to a clade of more complex eukaryotes sets up a kind of evolutionary event horizon beyond which we cannot see. The search for the origin of the eukaryotic nucleus therefore requires a journey deeper into our prokaryotic past.

### 4.2 An overview of theories of eukaryogenesis

It has long been postulated that the complex intracellular architecture of eukaryotes arose via endosymbiosis when one or more prokaryotic cells took up residence inside another cell (reviewed in [84]). In line with this hypothesis, early phylogenetic studies showed that the eukaryotic genome is composite in nature, including a strong alphaproteobacterial signal [85–89], together with a strong archaeal signal [90–95]. These bacterial and archaeal ancestors of eukaryotes appear to have made complementary contributions to the biology of the eukaryotic cell. As an example of this, the core mitochondrial machinery (e.g. mitochondrial rRNA, ribosomal proteins, together with many of the enzymes involved in electron transport) appears to be of alphaproteobacterial origin [96–100] (Figure 5). Conversely, the core information processing machinery, including that involved in DNA replication [101–104], chromatin structure [105–109], transcription [110–115], translation [116–122], and RNA processing [123, 124], along with cell division [125–127] (Figure 5), appears to be of archaeal origin. These data are consistent with the possibility that an alphaproteobacterial symbiont that gave rise to mitochondria was taken up by an archaeal host cell.

The discovery of the Lokiarchaeota (now Lokiarchaeia) and other members of the Asgard archaea (now Asgardarchaeota) in metagenomic assemblies from environmental samples [128–132], significantly strengthened the case for eukaryotes having emerged from an archaeal host. This is because phylogenetic analyses showed that Asgardarchaeota are the closest living archaeal relatives of eukaryotes (Figure 4). Furthermore, different Asgardarchaeota genomes were found to encode a wide range of so-called eukaryotic signature proteins (ESPs) – proteins whose homologues were previously thought to be specific to eukaryotes [128–130, 132]. Strikingly, these include cytoskeletal actin and several actin regulators [133, 134], the ESCRT membrane remodeling machinery, ubiquitin and its associated ligases and de-ubiquitinases [135], together with numerous small GTPases - most notably the Rags [128–130, 136, 137] (Figure 5). While some of these proteins have homologues amongst members of the TACK archaea and Euryarchaeota [80, 94, 138], these proteins are more widely distributed across the various Asgardarchaeota, where they often appear to be part of cellular machinery that is closer in terms of complexity and component parts to the corresponding eukaryotic machinery [130, 132, 139]. Many of these genes were likely present in the last common ancestor of Asgardarchaeota and eukaryotes, and they have likely played important roles in the evolution of cellular complexity during eukaryogenesis [128–130, 132].

**Figure 6:**
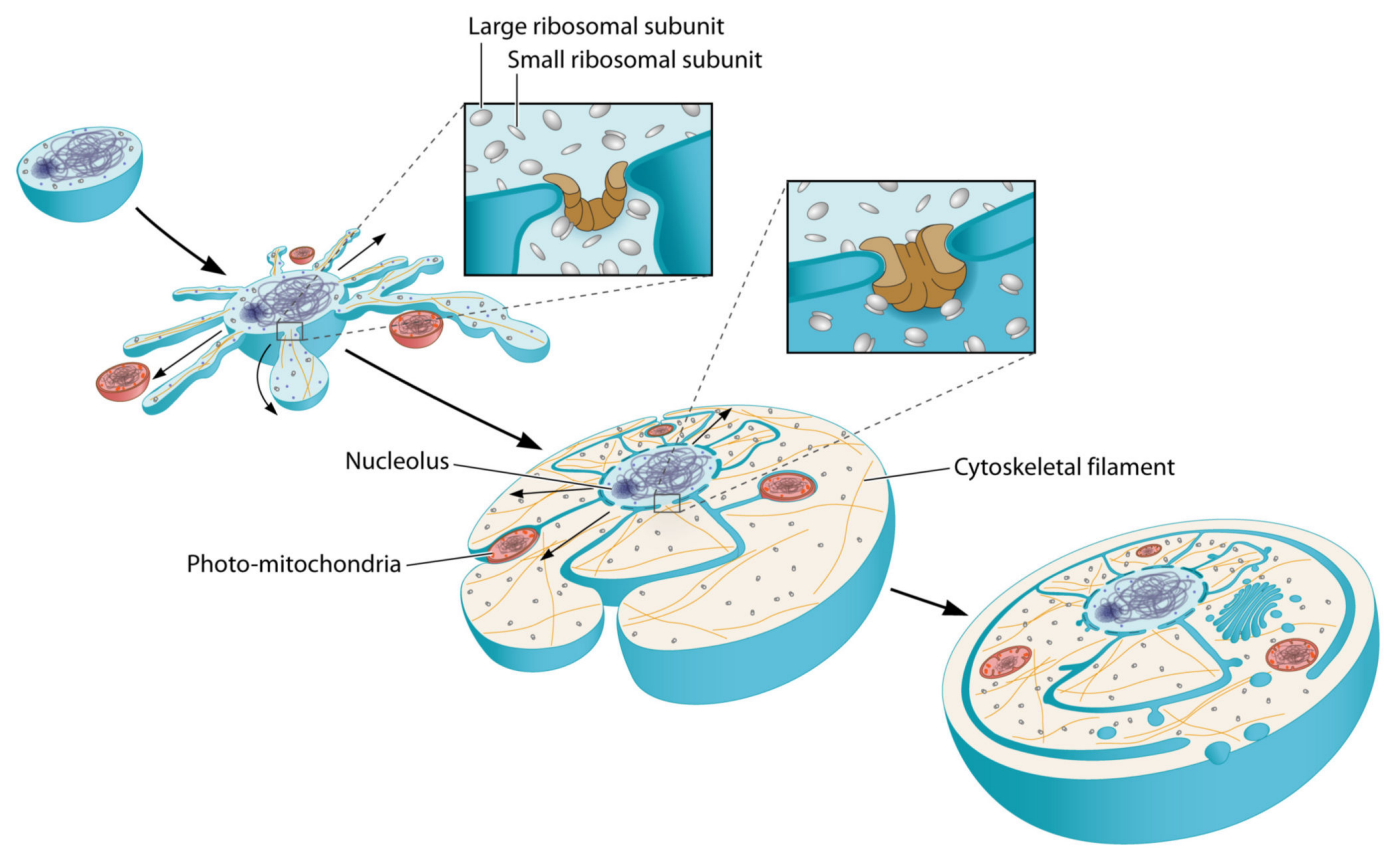
Diagram depicts the stepwise evolution of the eukaryotic cell as imagined under the inside-out model [5]. The cell in **Step 1** resembles a TACK archaeal cell, with a nucleolar-like structure where rRNAs are assembled into ribosomes, a single bounding membrane and a surface S-layer. The cell in **Step 2** has protrusions that make contact with bacterial partners (red), facilitated by a reduced S-layer. The internal space within these protrusions acts as a nascent cytoplasmic compartment, which is separated from the cell body by protrusion necks, where a region of high membrane curvature is stabilised by multimeric proteins that bind to the membrane from the inside. These structures function to confine the genome to the cell body. In **Step 3**, the separation of a nascent nucleoplasm and cytoplasm is enhanced by the duplication of the machinery at protrusion necks (brown in the inset) and by the onset of directional, energy-dependent trafficking across this boundary. As a result, RNAs are only translated upon entry into the cytoplasm (indicated in green in inset). The increased curvature induced by duplication of the machinery at the neck of protrusions, together with the emergence of proteins that encourage the self-association of membranes, force the membrane to fold back over the cell body, effectively insulating the cell body from the chemical and physical environment. The partial fusion of protrusions leads to the formation of a more continuous cytoplasm. Proto-mitochondria reside in the spaces in between neighbouring cytoplasmic compartments which are topologically equivalent to the lumen of the endoplasmic reticulum. Small black arrows indicate the flow of genetic information out from the centre in Steps 2 and 3. In the final step, **Step 4**, the formation of a plasma membrane by a process of self-engulfment (as a single cytoplasmic bleb wraps around the whole and undergoes a single membrane scission event (see also Figure 7)) yields a cell with a structure similar to that of a eukaryotic cell, with a topologically separate ER and plasma membrane, a continuous nuclear envelope-endoplasmic reticulum, and trapped mitochondria, which then enter the cytoplasm. For an in-depth description of the entire process see the original inside-out model.

**Figure 7:**
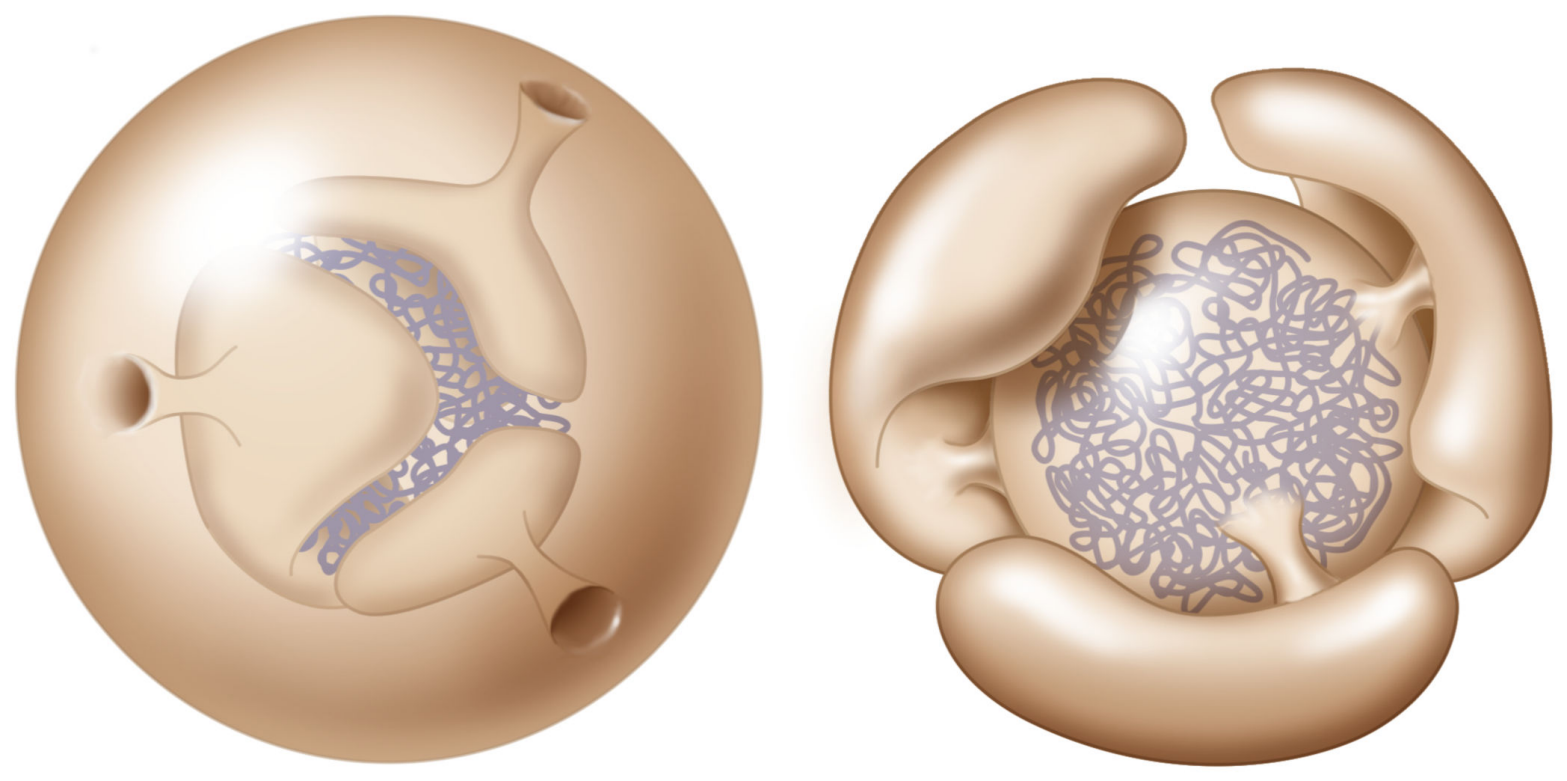
Diagram shows the intermediate steps in the process of eukaryogenesis expected under an **Outside-In model (left)** and an **Inside-Out model (right)**. Note that under the outside-in model, **left**, in order to generate a cell topologically similar to a eukaryotic cell (see Figure 6, step 4), multiple membrane remodelling events are required to fuse the separate endoplasmic reticulum (ER)-like compartments that form into a single lumenal network that is continuous with the nuclear envelope, and to generate a nuclear compartment connected to the cytoplasm via pores. In addition, additional membrane remodelling events are required to cut all the connections linking the nascent NE-ER compartment to the outer membrane to generate a topologically separate plasma membrane. By contrast, under the inside-out model shown in the diagram on the **right**, cells already possess a nascent nuclear compartment, a continuous ER and NE, and nuclear pores, but lack a single continuous cytoplasm and a plasma membrane. A topologically separate plasma membrane can be generated by a process of “auto-phagocytosis” whereby one protrusion extends around the whole, and undergoes a single scission event.

While these findings support the hypothesis that eukaryotes emerged from a partnership between at least one alphaproteobacterial ancestor [85], which gave rise to the mitochondria, and an asgardarchaeotal partner [80] (see Figures 4 and 5), the debate is far from settled. The reasons for this are severalfold. First, families of genes assigned to the last eukaryotic common ancestor (LECA) include representatives with diverse or unresolved prokaryotic origins [96–100, 140]. This has led some to postulate three or more partners contributing to the emergence of eukaryotes [8]. The difficulties in assessing such claims have been confounded by the fact that all alphaproteobacterial [141], asgardarchaeotal [129, 142, 143] and eukaryotic genomes [144–146] have been shaped by horizontal gene transfer events throughout their evolutionary history (see Figure 5). Furthermore, eukaryotic genomes are characterized by a large set of proteins and domains absent from prokaryotes, whose origins remain unknown [147, 148]. Finally, the current phylogenetic data provides few insights as to the origins of organelles, including the nucleus, and has done little to reveal the relative timing of organelle acquisition. Because of these uncertainties, the current data are compatible with aspects of different models put forward in an attempt to explain the origins of the eukaryotic cell (see Box 1, together with Figures 6 and 7).

Models of eukaryogenesis (see Box 1) exist on a continuum, but can be broadly divided into “mito early” scenarios, in which the acquisition of the alphaproteobacterial endosymbiont represents the first step on the path to the eukaryotic cell, and “mito-late” scenarios, which imagine mitochondria being acquired through a process akin to phagocytosis relatively late in the process of eukaryogenesis by a complex proto-eukaryotic host cell that already possessed a nucleus [80, 149]. In addition, models can be grouped by topology into “outside-in” versions, which propose that the nucleus was formed *de novo* following the coalescence of membranes around the DNA in a manner resembling nuclear reformation following exit from an open mitosis (Figure 7); models that imagine a third partner in the form of a virus [150, 151] or another cell [8] giving rise to the nucleus; and the “inside-out” model [5], in which the nucleus and ER are envisioned as having arisen from extensions of the original bounding membrane of an archaeal cell (Figure 6 and 7).

### 4.3 Nuclear origins in light of cell biological features of Archaea

The existence of very different models that all claim to explain the evolution of the various cellular features of eukaryotes might lead one to conclude that the origin of the nucleus is unlikely to be resolved anytime soon. However, by focusing on the eukaryotic membrane system and membrane trafficking, the plethora of models obscures the long-appreciated fact that many of the core functions of the nucleus are executed by proteins that have their origins in archaea (Figure 5). In fact, numerous studies over the past decades have characterized molecular machines in archaea that have close, if more complex, counterparts in eukaryotes, many of which function within the nucleus [101–127]. These parallels are most evident from molecular cell biology studies carried out using *Sulfolobus* cells - currently the most experimentally tractable member of the TACK [94] archaea; a sister group of the Asgardarchaeota and Eukarya [80]. This type of analyses has shown that *Sulfolobus* cells use close counterparts of the machinery found in eukaryotes to carry out many of the core information processing steps that are central to life including: DNA replication [102, 160], DNA-dependent RNA transcription [113], aspects of RNA processing [161], rRNA modification, ribosome assembly [162], messenger RNA processing [163], translation [120], protein secretion, protein degradation [164] and protein glycosylation [165]. In addition, as they grow and divide [166, 167], *Sulfolobus* cells pass through discrete G1, S, G2 and Division phases, which resemble phases of the eukaryotic cell cycle. Since *Sulfolobus* cells lack obvious homologues of the cell cycle clock (CDK/Cyclins), it is not yet known how they might regulate orderly passage through the cell cycle. However *Sulfolobus* cells express proteins involved in the regulation of transcription that possess a Cyclin-box fold and whose expression oscillates across the cycle [168]. In addition, proteasome-mediated degradation has been implicated in resetting the *Sulfolobus* cell cycle, just as it has been in eukaryotic cells [169]. Furthermore, *Sulfolobus* cells initiate S-phase [166] via the near synchronous firing of multiple replication origins using counterparts of the machinery used in eukaryotes, which include homologues of ORC/Cdc6, Cdc45, MCM and GINS [167]. Like eukaryotes, *Sulfolobus* cells also organize their genome into domains using SMC proteins [170], and express chromatin organizing proteins that, like histones (which are present in the vast majority of archaea including other members of the TACK but have been lost from *Sulfolobales* [106, 108, 171, 172]), are subject to regulation by acetylation [113] - including a chromatin protein Alba that has homologues in eukaryotes [173]. Thus, the parallels between the core information processing machinery present in TACK family archaea and the corresponding nuclear machinery present in eukaryotes are striking.

Despite the presence of homologs of proteins that function in the eukaryotic nucleus, there is no sign of TACK archaeal cells having anything like a nuclear envelope. With a few notable exceptions, including *Ignococcus*, which has two membranes and no S-layer [1, 174], most TACK archaeal cells described thus far have a single compartment bounded by a plasma membrane. These data could be used to argue that these archaeal relatives cannot tell us much about the origins of the nucleus. However, there is a way out of this conundrum. The difficulties of imagining how an archaeal cell might have given rise to a cell with a nucleus and cytoplasm come in part from the tendency to look at the nucleus as a distinct organelle that is separated from the cytoplasm by a double membrane. When the cytoplasm and nucleoplasm are viewed as sub-domains within a common compartment, as discussed above, it is easy to see the nucleus as having arisen via many small stepwise changes in the organization of a simple archaeal cell that resulted in the separation of information storage and processing activities (found in the modern eukaryotic nucleus) from protein synthesis and metabolism (activities usually confined to the eukaryotic cytoplasm). This idea plays a central role in the inside-out model of eukaryogenesis [5].

Under the inside-out model [5], the nucleus is suggested to have its origins in a simple archaeal cell (Figure 6, step 1) that develops protrusions (Figure 6, step 2). This cellular organization establishes spatially separate domains equivalent to a nascent nucleoplasm, where the DNA is housed, and a proto-cytoplasm, i.e. the site of protein synthesis, metabolism, and contact with the environment. In such a cell, machinery would likely be required to keep the genome out of protrusions, and to stabilize the sites of high local membrane curvature at junctions connecting protrusions to the cell body by preventing scission by the membrane remodeling ESCRT-III machinery, which induces membrane remodelling at topologically similar structures in archaea and eukaryotes [135]. Under the inside-out model, the elaboration of the machinery at the interface of the cell body and protrusions would have enabled the regulation of bi-directional traffic of large macromolecules (Figure 6, step 3), helping to prevent chromatin and intermediates in the information processing pipeline (like immature mRNAs and partially assembled ribosomes) from entering the nascent cytoplasm, i.e. protrusions. In this sense, the machinery at the necks of protrusions would resemble the nuclear pore complex and the diffusion barrier at the base of cilia in modern eukaryotes [5, 46].

If this view of early eukaryotic cell evolution is correct, the genomes of the Asgardarchaeota might be expected to hold clues to the origins of eukaryotic cell organisation. However, while asgardarchaeal genomes encode numerous homologues of proteins involved in eukaryotic information processing, cytoskeletal proteins, membrane remodeling complexes and regulators, the dynamic organisation of a cell is not something that can be deduced from genomic information alone [176]. A genome encodes the machinery required for growth and division rather than explicitly encoding cell biological features like cell size, structure or numbers of membrane bound compartments. As case in point, there is currently no simple way to analyze the genomes of *Sulfolobus acidocaldarius* and *Ignicoccus hospitalis* cells and say how many membrane compartments the two related cells possess (one and two, respectively). Thus, the availability of high quality genomes of Lokiarchaeia and other Asgardarchaota is insufficient to determine their cell biology [176]. To do so, one must observe cells under a microscope.

**Figure 8:**
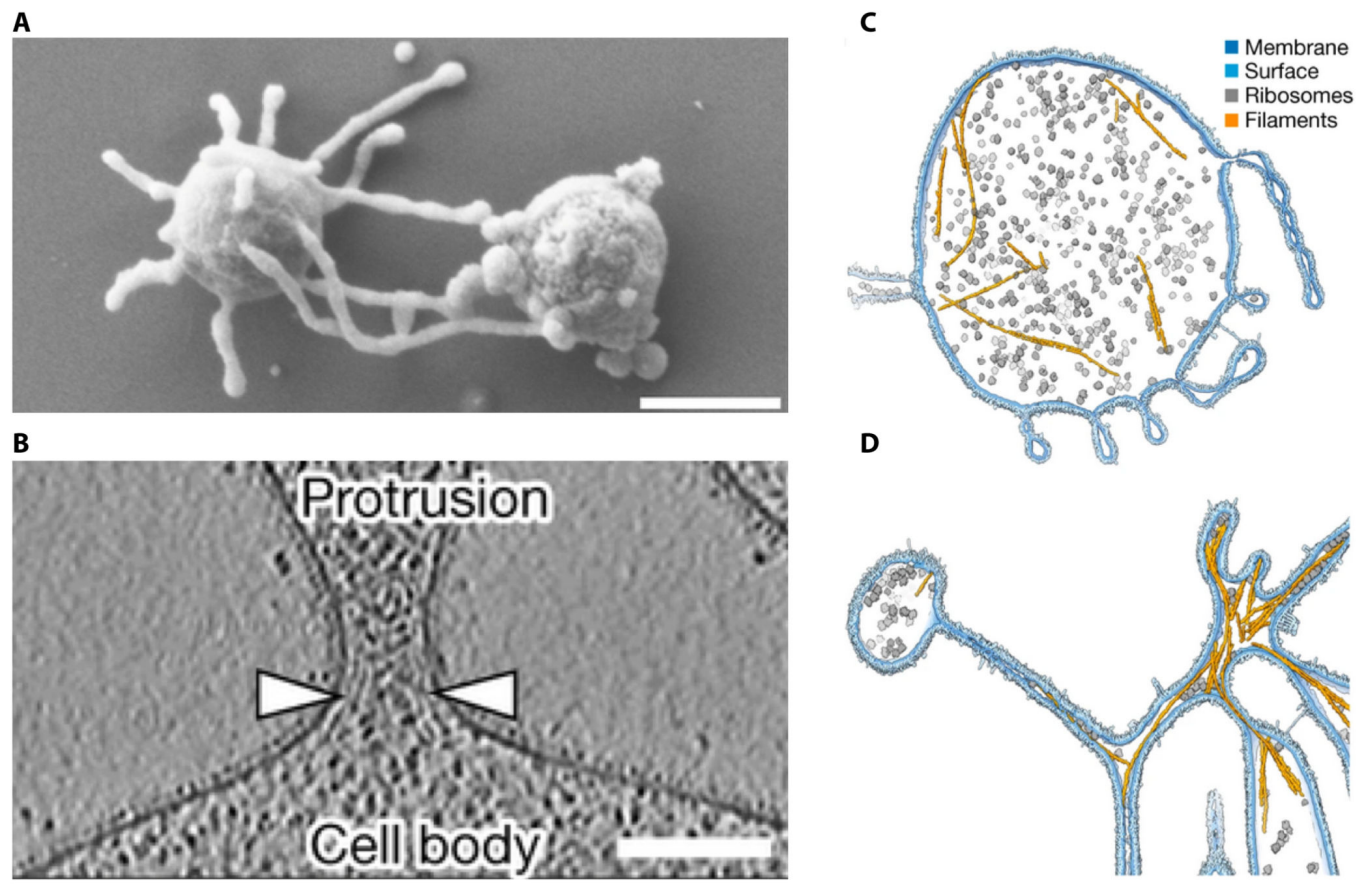
This figure, kindly reproduced with permission of the authors from Rodrigues-Oliveira et al, [175], shows electron microscopic images of Lokiarchaeia cells. **A**. An SEM shows a Candidatus Lokiarchaeum ossiferum cell (left) making contact with a possible syntropic partner via protrusions. **B**. A zoomed in CryoEM image shows the highly curved neck that separates the Lokiarchaeum cell body from its protrusions, and the electron dense layer that underlies it. **C and D** show Candidatus Lokiarchaeum ossiferum cells in which actin filaments (ochre), ribosomes (grey) and the bounding membrane have been imaged using CryoEM and highlighted in different colours.

This only became possible in 2020 once a representative of the Lokiarchaeia - *Candidatus* Prometheoarchaeum syntrophicum [175] - had been cultivated. This feat was 12 years in the making and was followed by the description of an enrichment culture of a second member of the Lokiarchaeia, *Candidatus* Lokiarchaeum ossiferum in 2023 [177]. Strikingly, the Lokiarchaeia enriched in the two studies closely resemble one another when imaged using electron microscopy. Both studies described small, micron-sized cells with long finger-like protrusions emanating from a spherical cell body [175, 177] (Figure 8), through which they were seen contacting other cells in the culture (Figure 8A). Significantly, the Lokiarchaeia in both studies also lacked internal membranes and anything resembling an internal nuclear compartment. These observations led to the proposition that Lokiarchaeia use protrusions to make contact with syntropic partners through which they can exchange metabolites [175], as was suggested as a first step towards eukaryogenesis under the inside-out model [5].

Furthermore, in line with this suggestion, when grown on organic compounds like amino acids, the growth of both cultivated representatives of Lokiarchaeia was found to depend on partner organisms to which they can transfer electrons in the form of hydrogen and formate – consistent with predictions from genomic analyses [178]. This type of syntrophic lifestyle has been hypothesized as a potential driving force leading to intricate interactions of the prokaryotic ancestors of eukaryotes [5, 8, 178–180].

While it is tempting to speculate, based on these data, that the asgardarchaeotal ancestor of eukaryotes was capable of syntrophic growth, it is important to note that Lokiarchaeia are only distantly related to the Hod and Heimdallarcheia, which currently appear to comprise the lineages most closely related to eukaryotes, and which have more diverse metabolic repertoires than Lokiarchaeia [130, 142, 178, 181]. Thus, gaining a more comprehensive view of the metabolic and cellular diversity of the Asgardarchaeota will require the enrichment and imaging of additional taxa.

### 4.4 Possible archaeal origins of nuclear functions

Given the archaeal origins of a large proportion of the information processing machinery present in eukaryotic cells, cell biological studies looking at the structure of Lokiarchaeia, and TACK archaea, like *Sulfolobus*, provide a useful starting point for thinking about the origin of the nucleus. Specifically, they clarify ways in which the regionalization of cellular space could have emerged, leading to the spatial separation of information processing events from translation and metabolism.

Several pieces of evidence already point to the possibility of some archaea having an ordered internal space, despite lacking distinct membrane-bound compartments. First, the genome in several TACK archaea appears physically confined to a small portion of the available cytoplasm, rather than being spread throughout the cytoplasm as it often is in bacteria. In *Sulfolobus acidocaldarius*, where this process has been studied using live cell imaging, the genome appears spread along a portion of the membrane in interphase [182]. Then, as cells prepare to divide, the genome appears to detach from the membrane as it compacts, leading to the formation of two discrete and separated DNA masses each of which is partitioned into one of the two daughter cells at cytokinesis [182]. This is not specific to *S. acidocaldarius*: the nucleioids of other *Sulfolobales* species as well as *Nitrosopumulus maritimus*, a member of the Thaumarchaeota, undergo similar changes in organisation across the cell cycle [183]. Superficially at least, this process resembles mitotic chromosome condensation in eukaryotes. Even more strikingly, in fluorescent images of Lokiarchaeia [177], the genome appears confined to the cell body and absent from protrusions – just as was envisaged for an early intermediate on the path to eukaryogenesis under the inside-out model (Figure 6). Conversely, while a few actin filaments were observed in the central body of these Lokiarchaeia using cryogenic-electron microscopy (cryo-EM), the majority of the signal generated using a fluorescently-labelled actin antibody was seen in protrusions [177]. These data suggest that Lokiarchaeia may possess a spatially distinct proto-nucleoplasm and proto-cytoplasm.

In eukaryotic cells, the spatial separation of cellular functions is enforced and amplified by diffusion barriers (at the nuclear pore and at the base of cilia); gradients in GTPase activity; protein and RNA trafficking via cytoskeletal elements; local translation; and the use of localization tags, such as nuclear localization signals. Therefore, it is important to determine whether similar factors exist in archaea. As we discuss below, preliminary data already suggest that archaeal cells possess simple versions of all five types of regulatory systems.

Taking each in turn:

#### 4.4.1 Diffusion barriers

Asgardarchaeota have membrane connections at the base of protrusions that are similar in width (^~^100nm) to the size of nuclear pores in eukaryotes [177]. In cryo-EM images, the membrane at the funnel-shaped necks of these protrusions appears to be bound by cytoplasmic protein density potentially indicating the existence of membrane-associated proteins that provide the highly curved membrane with structural stability (Figure 8B). This is one of the main functions of NPCs in eukaryotes [184]. At the same time, such proteins could function to limit the free diffusion of large macromolecular complexes, such as chromatinised DNA or RNPs, between the cell body and protrusions. Furthermore, proteins at the neck of protrusions may limit proteins to different parts of the continuous bounding cell membrane – as occurs in eukaryotes in the partitioning of proteins between the inner and outer nuclear membrane, and between the rough and smooth ER.

#### 4.4.2 GTPase gradients

While other Bacteria and Archaea, including TACK archaea and Euryarchaea, possess a number of small GTPases [137, 185], Lokiarchaeia and other Asgardarchaeota possess them in abundance [128, 129, 136, 137]. It is not clear why this might be. Thus far, there is little evidence that these archaeal small GTPases bind membranes or are modified by the covalent attachment of lipid moieties (with the exception of a few with transmembrane domains [176]). Nevertheless, it is possible that some of these GTPases function like Ran to organize intracellular space in conjunction with spatially separate GEFs and GAPs. Given that GTPases can act as diffusible switches, they may also enable regionalization of the single cytoplasmic space and/or the single bounding membrane.

#### 4.4.3 Cytoskeletal trafficking of protein and RNAs

Lokiarchaeia and other Asgardarchaeota possess close homologues of eukaryotic Actin and Tubulin [129, 133, 134]. In Lokiarchaeia, while actin filaments have been seen extending into protrusions [175, 177] it is not yet known if they help to generate and/or stabilize protrusions. In the available images, the actin filaments in Lokiarchaeia don’t appear highly organized or polarised with respect to one another or the membrane (Figure 8C). Nevertheless, *in vitro*, these filaments appear to be dynamic [133, 134]. This suggests the possibility that, in cells that lack clear homologues of actin-dependent molecular motors, the growth, shrinkage and treadmilling of such dynamic cytoskeletal polymers might generate flows that stir the cytoplasm and/or direct the transport of material associated with filament ends [186, 187]. This type of “cytomotive” behavior is used by eukaryotic microtubules to power the movement of chromosomes during mitosis. In cells with long protrusions where the diffusion of large complexes, such as mRNPs, to and from protrusion tips is likely to be very slow [188], such assisted diffusion may be critical.

#### 4.4.4 Local translation

Many archaea possess counterparts of ribosome processing machinery present in eukaryotes that are absent from bacteria [120]. These factors include SnoRNAs [189], homologues of Fibrillarin, and several Fibrillarin-associated proteins that act as rRNA methylases, all of which are key components of the eukaryotic nucleolus [162, 190]. This suggests the possibility that such proteins may function to ensure the spatial separation of active and inactive ribosomes in archaea. If these proteins and RNAs accumulate in spatially segregated domains, this may explain the physical separation of DNA and rRNA observed in Lokiarchaeia as imaged by Avci et al [191]. This type of regionalisation could be enhanced by other RNA processing enzymes. Archaeal cells (and many bacteria) possess a host of RNA processing enzymes [192] and RNA binding proteins that could function in this way. These include homologues of the enzymes that cleave the 3’ of RNAs, and DDX helicases, which induce local mRNA unwinding and translation in eukaryotes [31, 47]. Furthermore, there are archaeal homologues of Nmd3 [31, 47] [120] and TIF6/SBDS, which function to keep pre-ribosomes inactive in the nucleus in eukaryotes [193, 194]. It should be noted here that, although archaea possess type-II self-splicing introns [195], and RNA insertions (predominantly in rRNAs and tRNAs [71, 196]), there is no evidence of archaea possessing eukaryotic-like introns within protein coding RNAs [195]. Thus, for the moment, the evolutionary origins of the eukaryotic spliceosome [197, 198] appear complex and remain unresolved [197, 198]. Taken together, however, these data suggest the possibility that some archaea may be able to separate transcription and RNA processing from the site of translation, using a simplified version of the process taking place in eukaryotes.

#### 4.4.5 Short protein localization tags

NLS sequences in eukaryotic cells typically consist of short stretches of basic amino acids or a mixture of basic and surface-exposed hydrophobic amino acids. Similar sequences are present in ribosomal proteins in most archaeal groups, including DPANN and Euryarchaeota (all of which are thought to lack anything resembling a nuclear compartment [199]), where they likely function as rRNA binding motifs. NLSs are also present in eukaryotic proteasomes and some archaeal proteasomes [200]. These data suggest the possibility that NLSs may have originated in archaea as low affinity nucleic acid binding motifs that concentrate proteins in parts of the cell rich in rRNA or DNA, even though these cells lack a spatially distinct nucleus. Such sequences could have then been redeployed to direct nuclear/cytoplasmic traffic with the advent of a complex nuclear pore. By the same argument, other small motifs used to direct different types of cellular traffic in eukaryotic cells may have counterparts in archaea that function to locally concentrate specific proteins in the context of a single compartment.

## 5 An updated model for an inside-out origin of the eukaryotic nucleus

Bringing these arguments and data together, we propose a stepwise path for the evolution of different nuclear functions building upon the inside-out model of eukaryogenesis. This is detailed below and depicted in Figures 6 and 7.

### Step A: The separation of transcription and translation

The starting point for the model is a structurally simple early archaeal ancestor of eukaryotes that lacks internal membranes, but which possesses machinery that enables the partial physical separation of transcription and translation for some transcripts. We propose that this separation is achieved via several processes working in tandem. First, rRNA processing and modifications (including Fibrillarin-dependent methylation [162]) are spatially confined to sites of rRNA transcription through the establishment of a phase-separated region of the cytoplasm that acts like a primitive nucleolus [201]. Second, the delayed completion of ribosome assembly ensures that a subset of ribosomes remain inactive until meeting an RNA substrate at a relevant site in a cell (as occurs in eukaryotes for secreted proteins as a result of SRP14-mediated inhibition of translation [202]). Third, some newly transcribed and processed RNAs [203] are rapidly assembled into inactive RNPs, preventing their association with active ribosomes or delaying the completion of translation [204] until they reach the correct cellular site. As a result, the translation of some RNAs is contingent on RNP disassembly driven by specific local RNA helicases [205].

### Step B: The separation of a nascent cytoplasm from a nascent nucleoplasm

In a more recent common ancestor of Asgardarchaeota and eukaryotes, this partial separation of transcription from translation is augmented by the spatial confinement of these processes to the cell body and protrusions. This is facilitated by machinery localised at the necks of protrusions (Figure 6, step 2). Protein assemblies, like those visible in the electron dense material underlying curved membranes in electron micrographs of Lokiarchaeia cells [177] (Figure 8), function to prevent the movement of chromatin into metabolically active protrusions. Later in the process of eukaryogenesis, the elaboration of this machinery at protrusion necks acts to bias the free diffusion of large macromolecules (e.g. cytoskeletal polymers and RNPs). Together, this machinery facilitates the spatial differentiation of the cell into a metabolically inactive central domain (a proto-nucleoplasm) in which nucleic acids are stored and processed, and peripheral protrusions (a protocytoplasm), where active metabolism occurs and which act as sites at which physical contact with symbionts is established - enabling the efficient transfer of electrons and/or substrates between partners [175]. Over time, the evolution of machinery accentuating local differences in the accumulation of proteins in different parts of the cell enhances regional specialization of the single bounding membrane and cytoplasm.

In cells with long thin protrusions, diffusion is slow. This both limits the coordination of distant cell biological processes [206, 207] (Figure 6, step 2) and facilitates the separation of activities, like metabolism and information storage, which might otherwise interfere with one another. In such a diffusion-limited system, we imagine cells using dynamic cytoskeletal filaments that bind and hydrolyse NTPs, like those present in Lokiarchaea, to regulate the flow of genetically encoded information between regions, by coupling the unidirectional transport of large macromolecular complexes to discrete phases of filament growth or shrinkage and/or by stirring the cytoplasm. Note that the distance over which such cytomotive filaments [208] operate depends on their persistence length, which is determined by the structure of the polymer [209], so that any increase in cell size will need to be accompanied by an increase in filament stiffness, e.g.via filament bundling or microtubules. Such cytomotive forces will be especially important for directing the traffic of large, slowly diffusing particles, like pre-ribosomes or RNPs to the tips of protrusions (note a 25kDa protein is encoded for by an RNA of ^~^200kDa). Over time, we imagine enzymes emerging to bias these movements. For example, homologues of the rRNA remodeling complex Midasin [210] − an ATPase involved in the activation of translation [211, 212] and related to eukaryotic Dynein [213, 214] − may aid this process, setting the stage for the later emergence of ATP-hydrolyzing cytoskeletal molecular motors.

### Step C: Formation of a double nuclear envelope

At the next stage of eukaryogenesis (Figure 6, step 3), we imagine protrusions expanding and folding back onto the surface of the cell body to generate a double nuclear envelope. This change in membrane organization would have been aided by the evolution of complementary surface adhesive proteins, perhaps similar to the glycosylated SUN and KASH proteins [31, 47], which in eukaryotes function to hold inner and outer nuclear membranes together [5], and by the duplication of the machinery supporting the curved membrane at protrusion necks (Figure 6, step 3). This is depicted in the model as a switch from a half-pore with 8-fold rotational symmetry to a full nuclear pore with an additional axis of symmetry. This change in cellular organization, in which the peripheral proto-cytoplasm and membrane wrap the proto-nucleus, physically isolates the cell body housing the genome from the external environment [5]. The duplicated pore can then also participate in the regulation of nuclear-cytoplasmic transport, perhaps by coupling movement across the neck of protrusions to a Ran-GTP’ase-like system, like that described above. In this case, GTP hydrolysis could power the directional transport of material into or out of the nascent nuclear compartment, where the genome resides. Together, these innovations enforce a more complete separation of transcription, ribosomal assembly and RNA processing, from active protein synthesis. This sets the stage for the evolution of splicing, perhaps using components derived from Asgardarchaeota, Alphaprotebacteria and other Bacteria [195]. At division, we envision the partial opening of the curved necks at the base of protrusions (equivalent to a semi-open mitosis [11]) facilitating the fair mixing of proto-cytoplasm and proto-nucleoplasm as a prelude to the even segregation of nuclear and cytoplasmic parts at division.

### 5.1 The role of symbiosis in the origins of nuclear functions

Having outlined a possible scheme above for the stepwise evolution of a nuclear-like compartment, it is worth emphasizing that our focus here on the archaeal host and earliest stages of eukaryogenesis deliberately leaves aside the role of the symbiotic partnership with the alpha-proteobacterial cell that gave rise to the mitochondria, which was covered in detail in the original inside-out model [5]. It is clear, however, that the mitochondrial ancestor contributed to the selective pressure driving the emergence of certain cellular features, and played a critical role in the evolution of eukaryotic cell organization [6–8, 80, 90] (Figure 5). For instance, the transfer of genes from bacterial partners to the archaeal host nucleus [179], together with horizontal gene transfer from other bacteria [8, 140, 215], likely contributed to the expansion of the nuclear genome, to changes in translation required prevent crosstalk between the host and mitochondrial ribosome assembly pathways, to the emergence of introns, and to the acquisition of new metabolic functions. This latter process included the synthesis of fatty acids and sterols [216–218], leading to a switch in membrane composition that is likely to have facilitated the acquisition of dynamic membrane trafficking [5]. The advantage of putting these aspects of the inside-out model aside in this discussion of the origin of the nucleus, is that it allows us to propose a framework that can be explored by experiment through the study of existing cultures of TACK archaea and Asgardarchaeota. Thus, while this extension of the original inside-out model is speculative, it suggests a simple series of steps that could have led to the emergence of a proto-eukaryotic cell with a functioning nucleus and nuclear envelope in a way that does not necessarily depend on the prior emergence of mitochondria and a sophisticated vesicle trafficking system.

### 5.2 Experimentally testable questions raised by the model

In order to test the validity of this model for the origin of the nucleus it is important to improve the cell biological analysis of TACK and Asgard archaea, with a focus on studies exploring the path of protein synthesis and regionalisation of the cytoplasm and membrane. This should include experiments to determine:

1. How are protein-coding transcripts in TACK and Asgardarchaeota processed prior to translation [192]?
2. Is there a temporal delay and/or physical separation between transcription and translation in some cases in TACK and Asgardarchaeota?
3. Are pre-ribosomes assembled in a nucleolus-like structure in members of the TACK and Asgardarchaeota [201], and are there mechanisms to ensure that ribosomes become activated at specific sites far from their assembly?
4. Do different subsets of RNAs accumulate in the cell body and protrusions of Lokiarchaeia prior to the completion of translation? Do Lokiarchaeia express transcripts whose translation is inhibited by RNA binding proteins before being locally activated by RNA helicases?
5. Is genomic DNA excluded from Lokiarchaeia protrusions? If so, how?
6. Do proteins at the curved base of Lokiarchaeia protrusions serve as barriers to the diffusion of large protein and/or RNA complexes? Do these proteins also block the ESCRT-III mediated scission of protrusion necks?
7. Does the cytomotive activity of cytoskeleton polymers in Asgardarchaeota aid facilitated transport in diffusion-limited protrusions?
8. Do structures at the base of protrusions in Lokiarchaeia open prior to cell division to enable the mixing of different domains as an aid to division symmetry?
9. Do short localization tags, including NLS sequences, aid the local accumulation of proteins at specific sites within the cytoplasm or at specific membrane domains in Lokiarchaeia?
10. Do small GTPases help establish distinct domains of activity across lokiarchaeial cells?

### 5.3 Key challenges faced by the model

With the caveat that extant representatives derived from the asgardarchaeotal sister-lineage of eukaryotes have evolved for as much time as the eukaryotic lineage, future experiments could also turn up data that would be harder to reconcile with the model of nuclear evolution put forwards here; and thereby favour alternative models of eukaryogenesis. These include the following:

1. The demonstration of direct coupling between transcription and translation of all protein-coding transcripts in the closest archaeal relatives of eukaryotes.
2. Failure to demonstrate that Asgardarchaeota are able to establish and maintain locally distinct domains where specific RNAs, proteins and localisation tags accumulate.
3. The identification of close archaeal relatives of eukaryotes that possess a complex and dynamic internal membrane organisation (emanating from the cell surface (as shown in Figure 7, left) or the mitochondria), but which are not able to physically separate the genome and early information processing events (transcription and ribosome assembly) from protein synthesis and metabolism.
4. The identification of close archaeal relatives of eukaryotes that use coatamer-like assemblies to regulate vesicle trafficking but which lack anything resembling nuclear pores.
5. The identification of close archaeal relatives of eukaryotes that cannot physically separate early information processing events from protein synthesis and metabolism, but which are capable of phagocytosis.
6. The identification of symbiotic consortia consisting of bacterial cells that act as hosts to intracellular archaeal symbionts related to the asgardarchaeotal ancestor of eukaryotes – as envisioned in the syntropy model.
7. The identification of close archaeal relatives of eukaryotes that possess viral assembly factories that resemble membrane-bound nuclei.

## 6 Conclusion

In this hypothesis article we propose that our closest living archaeal relatives, TACK and Asgardarchaeota, hold clues to the early stages in the origin of the nucleus. This model builds on decades of molecular and cell biological evidence from studies using TACK archaea, like *Sulfolobus*, and on a very limited number of cell biological studies in Asgardarchaeota [219], which together suggest that, prior to the association of a mitochondrial symbiont, archaeal ancestors of eukaryotes already possessed a nucleolar-like domain and a cytoplasmic-like compartment in the form of protrusions that exclude DNA [177]. These data support the inside-out model [5] and imply that archaea can differentiate a single continuous cytoplasm and membrane into distinct genome-storage and metabolically active domains – just as eukaryotic cells do through the establishment of a nuclear-cytoplasmic compartment boundary. Bringing these data together, we propose that an array of biochemical functions that are associated with eukaryotic nuclei first arose archaea. These were then elaborated during eukaryogenesis, alongside changes in cell shape, to give rise to the nucleus. Under this model, the nucleus has its origin in archaea.

## Box 1

**Brief overview of select models proposed as explanations for the origin of the nucleus**

Models of eukaryogenesis can be divided into those that imagine the nucleus having been acquired via symbiosis and those that imagine it having arisen by the remodeling of an ancestral cell. We refer readers to comprehensive reviews [6–8, 80, 90] for an in-depth discussion. Note that all models have to contend with: i) the phylogenetic data, ii) the acquisition of alphaproteobacterial-derived mitochondria with two membranes, iii) the acquisition or gradual evolution of a nucleus, iv) eukaryotic cell organization (the presence of a continuous nuclear envelope and ER, and a topologically separate plasma membrane), and v) the fact that while much of the core protein machinery underlying eukaryotic cell organization appears to have its origins in archaea, eukaryotic membranes are largely constructed from bacterial-type lipids, and host both bacterial-derived respiratory chains (e.g. in the mitochondrial membrane) as well as important transmembrane proteins with archaeal origins (e.g. the translocon) (Figure 5).

**Endosymbiotic nuclear origin**

1. *Tripartite hydrogen and sulfur-transfer-based model (syntrophy)*. The syntrophy model proposes that the nucleus derives from an endosymbiotic archaeon that was engulfed by a sulfate-reducing deltaproteobacterial host cell, which also internalized a facultatively aerobic sulfide-oxidizing alphaproteobacterial cell that gave rise to mitochondria [8]. A strength of the model is that it can explain why it is that eukaryotic cells possess bacterial rather than archaeal-type lipid membranes. A weakness of the model is its failure to explain how the proposed requisite changes in membrane organization occured. First, it is not clear how the archaeon might have entered the cytoplasm of a bacterial host. Second, under the model, the bacterial host formed membranes around this central archaeal cell via the ingathering of membranes; as it is in classical outside-in models. As explained in Figure 7, this requires extensive membrane remodelling both to seal invaginating membranes, and to form a nuclear compartment that contains pores and is continuous with the endoplasmic reticulum. Third, the model proposes that the archaeal membrane was then somehow lost. This would have induced the mixing of deltaproteo¬bacterial and archaeal ribosomal machinery within a single compartment, likely compromising translation. In this regard, it is worth noting that eukaryotic ribosomes seem to be derived from archaeal, alphaproteobacterial, and, in the case of plastid-containing eukaryotes, cyanobacterial homologs, without strong indications of the existence of an additional prokaryotic source. Finally, the model proposes that one of the two outer membranes of the deltaproteobacterial host was lost and the ER internalized. The problem here is that this could not have been achieved in any simple way. Remove the outer membrane, and the ER lumen now becomes continuous with the environment. Remove the inner membrane, and the ER is lost and, more problematically, the intra-membrane luminal space fuses with the cytoplasm.
2. *Viral origin hypothesis*. This model proposes that the nucleus arose from a DNA virus replication factory in an archaeal cell, which also hosted the alphaproteobacterial endosymbiont [150, 151]. A key feature of this model is its focus on explaining evolution of the separation of transcription and translation, given that both archaea and bacteria tend to lack the machinery that generates and reads the 5’ m7G cap characteristic of eukaryotic mRNAs [152]. A key problem with this model is that the double membrane compartments viruses generate to allow RNA processing arise via remodeling of ER membranes and membranes of other internal organelles [153, 154]. Thus, a pre-requisite for this model to work is a pre-existing dynamic endomembrane system – something that all other models of eukaryogenesis aim to resolve. It is also not clear where traces of this virus are to be found in eukaryotic genomes [155].

**Autogenous nuclear origin**

1. *Outside-in model:* This model proposes that the nucleus arose as a result of the cytoplasmic membranes of a host cell, usually an archaeon, being recruited around the DNA in a manner resembling the exit from an open mitosis. Such internal membranes would, in turn, have arisen via internalization of the plasma membrane (Figure 7) via a process akin to the initial steps of endocytosis/phagocytosis [156]. Note that extensive membrane fusion is required to generate a single membrane-bound nuclear compartment that is connected to the rest of the cells via pores, and to generate a single ER network (Figure 7). At the same time, multiple membrane fission events are required to topologically separate the plasma membrane from the internal membranes (Figure 7).
2. *Mitochondrial derived membrane model*: Under this model, the nucleus arose from the extrusion of membranes from the outer surface of the mitochondria [157] via the coalescence of internal membrane vesicles - similar to that envisaged by outside-in models. The strength of the model is its ability to explain why the membranes of eukaryotic cells appear similar to those of alphaproteobacteria. The model also takes inspiration from the observation that vesicles have been seen budding off the outer membranes of gram-negative bacteria. However, the model doesn’t explain many specifics of eukaryotic cell architecture. For example, while vesicular trafficking of this type is used by eukaryotic cells to move lipids between organelles like the ER, Golgi and plasma membrane, it is not used to move lipids to or from the mitochondria as proposed. Instead, the movement of lipids between mitochondria and the ER in eukaryotes is mediated by proteins channels at membrane contact sites. In addition, it is hard to understand how nuclear pores arose under this model.
3. *Inside-out model*: Under the inside-out model, the first step in eukaryogenesis was the gradual emergence of distinct nuclear and cytoplasmic compartments. This was initiated when simple archaeal cells that housed their genomes in a cell body began to interact with bacterial partners in their environment via protrusions [5, 46] (Figure 6 and 7). Under the model, the sites at which these protrusions emerged from the cell body gave rise to nuclear pores, which became elaborated to control directional traffic between the nascent nucleoplasm and cytoplasm (Figure 6 step 3). After the acquisition of membrane remodelling activities and the growth of protrusions, a topologically separate plasma membrane was formed via a membrane scission event (Figure 6 step 4). The model also envisages protrusion-mediated contact between syntropic partners led to a gradual process of symbiotic integration, as bacterial genes for lipid biosynthesis were taken up by the archaeal host genome [5, 46]. This model has the advantage of requiring very few membrane remodelling steps to generate a cell that is topologically equivalent to the eukaryotic cell. The model also proved prescient in predicting the existence of archaeal cells that lacked internal compartments but which might interact with partners via protrusions (Figure 8). A perceived weakness of this model is that it requires a switch from archaeal to bacterial lipids, which was envisaged to have taken place during the long partnership between the bacterial cell and its archaeal host. While speculative, recent findings have shown that bacteria with mixed membranes exist in nature [158] and that E. coli cells with an engineered hybrid heterochiral membrane are viable [159]. In addition, it is clear that the eukaryotic counterparts of archaeal proteins that once resided in archaeal membranes, like Sec61, made the transition during eukaryogenesis and now perform a similar function in eukaryotic membranes. Taken together, these data suggest that a transition period with a mixed membrane poses less of a problem to models of eukaryogenesis than previously thought.
